# Estimation of Phosphorus and Nitrogen Waste in Rainbow Trout (*Oncorhynchus mykiss*, Walbaum, 1792) Diets Including Different Inorganic Phosphorus Sources

**DOI:** 10.3390/ani11061700

**Published:** 2021-06-07

**Authors:** Maria Consolación Milián-Sorribes, Ana Tomás-Vidal, David S. Peñaranda, Laura Carpintero, Juan S. Mesa, Javier Dupuy, Andrés Donadeu, Judit Macías-Vidal, Silvia Martínez-Llorens

**Affiliations:** 1 Aquaculture and Biodiversity Research Group, Institute of Animal Science and Technology, Universitat Politècnica de València, Camino de Vera 14, 46071 València, Spain; mamisor@etsia.upv.es (M.C.M.-S.); atomasv@dca.upv.es (A.T.-V.); dasncpea@upvnet.upv.es (D.S.P.); 2 R&D Department, Global Feed, S.L.U., Tervalis Group, Av. Francisco Montenegro s/n, 21001 Huelva, Spain; laura.carpintero@tervalis.com (L.C.); juan.sebastian@tervalis.com (J.S.M.); javier.dupuy@tervalis.com (J.D.); andres.donadeu@tervalis.com (A.D.); judit.macias@tervalis.com (J.M.-V.)

**Keywords:** inorganic phosphorus source, monoammonium phosphate, monosodium/monocalcium phosphate, phosphorus digestibility, rainbow trout

## Abstract

**Simple Summary:**

Aquaculture effluents with high levels of phosphorus (P) and nitrogen (N) contribute to eutrophication in the aquatic ecosystem. The environmental impact of phosphorus and N aquaculture waste may be diminished by modifying diet ingredients that improve phosphorous (P) digestibility, and therefore, reduce the P in metabolic waste. The content of P in fishmeal is high (30 g/kg), but the inclusion of fishmeal in the diet is reducing due to its high costs and limited accessibility; therefore, the addition of an inorganic P source is necessary to ensure a satisfactory level of available P in fish diets. Consequently, the present study aimed to evaluate the effect of four different inorganic P sources on P digestibility and excretion in rainbow trout (*Oncorhynchus mykiss*), as one of the most relevant aquaculture species. Monosodium/monocalcium phosphate with 2% of sodium source presented a P digestibility similar to monoammonium phosphate, but with lower nitrogen and phosphorus excretion into the environment, which is advantageous from a nutritional, environmental and industrial point of view (biofilters and recirculation systems in fish farms).

**Abstract:**

This study was conducted to evaluate the apparent availability and P and N excretion in rainbow trout (*Oncorhynchus mykiss*) using different inorganic phosphorus sources. With this goal, fish (153 ± 14.1 g) fed four inorganic P sources were assayed: monoammonium phosphate (MAP, NH_4_H_2_PO_4_), monosodium/monocalcium phosphate (SCP-2%, AQphos+, NaH_2_PO_4_/Ca(H_2_PO_4_)_2_·H_2_O in proportion 12/88), monosodium/monocalcium phosphate (SCP-5%, NaH_2_PO_4_/Ca(H_2_PO_4_)_2_·H_2_O in proportion 30/70) and monocalcium phosphate (MCP, Ca(H_2_PO_4_)_2_·H_2_O). Phosphorus (P) digestibility, in diets that included MAP and SCP-2% as inorganic phosphorus sources, were significantly higher than for SCP-5% and MCP sources. In relation to the P excretion pattern, independent of the diet, a peak at 6 h after feeding was registered, but at different levels depending on inorganic P sources. Fish fed an MAP diet excreted a higher amount of dissolved P in comparison with the rest of the inorganic P sources, although the total P losses were lower in MAP and SCP-2% (33.02% and 28.13, respectively) than in SCP-5% and MCP sources (43.35% and 47.83, respectively). Nitrogen (N) excretion was also studied, and the fish fed an SCP-5% diet provided lower values (15.8%) than MAP (28.0%). When N total wastes were calculated, SCP-2% and SCP-5% showed the lowest values (31.54 and 28.25%, respectively). In conclusion, based on P and N digestibility and excretion, the SCP-2% diet showed the best results from a nutritional and environmental point of view.

## 1. Introduction

Aquaculture is still a sector with a great growth potential, with improvements in fish feed formulations being essential to achieve a greater expansion. On the other hand, it is also necessary to promote a more sustainable production, reducing waste and environmental pollution [1], without affecting the nutritional attributes, quality and cost-effectiveness. Therefore, environmental management of intensive aquaculture is essential for the achievement of sustainable aquaculture in the coming years.

With this aim, a decrease in phosphorus (P) and nitrogenous (N) discharge from aquaculture effluents would reduce water eutrophication, and therefore, its environmental impact. The main strategy to achieve this reduction is through the optimization of diet formulation. P is a macromineral component of nucleic acids and cell membranes in living beings, also participating in energy processes [2,3]. Additionally, it plays an essential role in the formation and maintenance of bone structures, as well as the synthesis of other tissues such as muscle mass [4]. Despite the fact that fish can absorb minerals from water through their gills, fish require an additional source of P in their diet [5], since P is usually a limiting mineral in most natural waters, and its absorption rate from water is low.

P in fishmeal, mainly in the form of hydroxyapatite or bone phosphate as well as inorganic supplements, is relatively available to rainbow trout (*Oncorhynchus mykiss*). In contrast, approximately two-thirds of the P in plant sources is bound to phytate, being only partially available to fish [6], presumably due to low levels of intestinal phytase. On the other hand, the scarcity of fishmeal production and its high price is limiting its inclusion in the feed, it being substituted by plant protein sources [7,8,9,10]. Therefore, due to the greater inclusion in the diet of plant protein sources and their low P digestibility, the inclusion of an inorganic P source in feed is required. However, previous studies in common carp (*Cyprinus carpio*) production [11] reported reduced environmental effects in diets with high plant protein levels when the P dietary level is optimum. Therefore, the selection of the inorganic P source is an important issue and it will be based on its solubility and digestibility [12], which may be affected by the calcium level in the diet, as well as changes in pH under gastrointestinal conditions [13]. In general, monobasic phosphates from monovalent cations are more digestible and soluble, followed closely by monocalcium phosphate (MCP) and, beyond this, by tricalcium phosphates or bone apatite. This is generally applicable to marine and freshwater fish with a stomach, such as rainbow trout [14]. The combined use of phytase with a highly digestible inorganic P source, such as monosodium phosphate (MSP) and monoammonium phosphate (MAP), to meet a species’ P requirement might be an accurate choice to optimize P retention efficiency, and thereby, to reduce P excretion into the environment [15]. Moreover, the dietary supplementation of an organic acid mixture enhanced the growth and nutrient efficiency of dicalcium phosphates [16].

**Figure 1:**
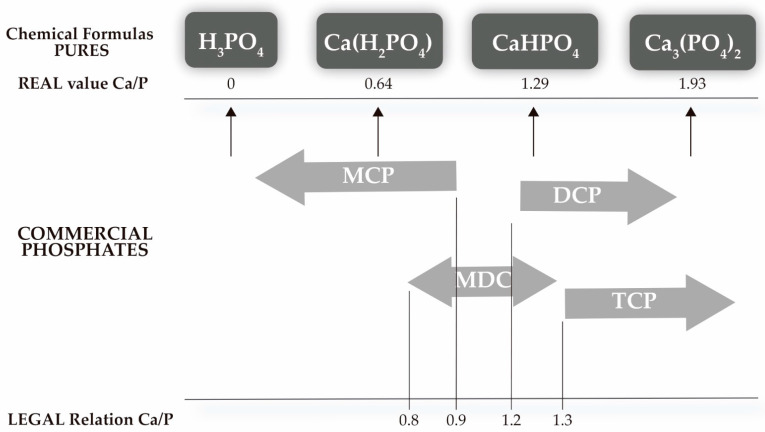
Characterization of commercial phosphates following the European Union Regulation [18] following the proportion of monocalcium phosphate (MCP), dicalcium and tricalcium phosphate (DCP and TCP, respectively) and phosphoric acid [20].

A low absorption and retention of nutrients will mean a higher discharge into water, affecting the sustainability of the production. Aquaculture effluents with high levels of P and N contribute to the pollution of the aquatic ecosystem through the eutrophication of natural fresh water. Consequently, aquaculture faces a dilemma: feed must meet P levels, but at the same time, feeding practices must comply with environmental guidelines to minimize the P load in the aquatic environment [17]. Inorganic commercial phosphates, under European Union Regulation [18], can be easily found on the market, being categorized according to the Ca/P ratio (Figure 1). This is due to commercial phosphates coexisting without a defined chemical composition, in the case of mixtures of monocalcium phosphate (MCP), dicalcium phosphate (DCP), phosphoric acid, carbonate and impurities. This undefined chemical composition is explained by the appearance of unwanted collateral reactions, events difficult to avoid at the industrial scale. This compositional variability causes different degrees of solubility in both water and citric acid, as well as variations in pH and the presence of other minerals. The mixture of chemical products ends up directly influencing the degree of bioavailability of the phosphate species in each species, which is defined as the degree to which a nutrient ingested from a particular source is absorbed and remains available for the animal’s metabolism [19].

Studies on rainbow trout [20] have stated that both MAP and MSP have higher levels of apparent P digestibility (≈90%) compared to other inorganic phosphates, such as MCP. Likewise, a recent study [21] reported an improvement in nutrient digestibility, mineral bioavailability and immune functions of MAP, monopotassium phosphate (MKP) and MSP when compared with MCP in high plant ingredient-based diets. On the other hand, from an environmental point of view, it should be considered that MAP can release a greater amount of non-protein nitrogen (N) into the water, probably as undigested ammonia through the feces released by fish [22].

In conclusion, the aim of the present study was to evaluate in vivo the P availability and excretion level of diets including four different inorganic P sources in rainbow trout. As a novelty, new formulations of inorganic phosphates were assessed from nutrition and environmental point of view for its inclusion in fish diets.

## 2. Materials and Methods

### 2.1. Fish, Facilities and Rearing Conditions

Around 200 rainbow trout with an average weight of 130 g were moved by an authorized service from the fish farm “El Zarzalejo” located in Zamora (Spain) to the Fish Nutrition Laboratory belonging to ICTA-UPV (Valencia, Spain). Before starting the feeding assay, all fish were acclimated to indoor rearing conditions for two weeks and fed a standard diet for rainbow trout (45% crude protein, CP; 22% crude lipid, CL; 6.8% ash; 0.81% Ca and 1% phosphate, Biomar Iberia S.A., Dueñas, Spain).

After 15 days of acclimation, 16 trout (153 ± 14.1 g) were redistributed into cylindric-conical tanks of 200 L with sedimentation columns based on the Guelph system [23] (four replicates per treatment). Afterwards, the fish were acclimated to new tanks for two weeks being fed once per day with a control diet (10:00 a.m.). The rest of the rainbow trout were housed in 4000 L tanks with an open water circuit, as a preventive measure.

In order to ensure the correct functionality of the facility and animal welfare, physic-chemical water parameters and mortality were checked daily. The oxygen concentration in the water and the temperature were monitored using a portable oximeter (Oxyguard Handy Polaris, Farum, Denmark). pH, ammonium and nitrites concentrations were analyzed with a pH meter and colorimetric test (MERK, Darmstadt, Germany), respectively.

The environmental conditions were as follows: salinity: 0 g/L, temperature: 16.10 ± 1.06 °C, oxygen: 8.04 ± 1.41 mg/L, pH: 7.9 ± 0.32, ammonium and nitrites: 0.0 mg/L and nitrates: 15 ± 2.3 mg/L. The photoperiod was natural (May to July). The duration of the experiment was 120 days. All tanks had aeration supply. The water temperature was kept constant thanks to the fact that the freshwater was taken from a freshwater well.

### 2.2. Experimental Diets

Four supplemental inorganic P sources were chosen for determining the apparent P availability and non-fecal P excretion: monoammonium phosphate (MAP, NH_4_H_2_PO_4_, Yara S.L., Helsingborg, Finland), monosodium/monocalcium phosphate (SCP-2%, AQphos+, Global Feed, S.L.U., Huelva, Spain), monosodium/monocalcium phosphate (SCP-5%, Global Feed, S.L.U., Huelva, Spain) and monocalcium phosphate (MCP, Ca(H_2_PO_4_)_2_·H_2_O, Global Feed, S.L.U., Huelva, Spain). Chemically, SCP-2% and SCP-5% were mixtures of monosodium and monocalcium phosphates, being NaH_2_PO_4_/Ca(H_2_PO_4_)_2_·H_2_O at a proportion of 12/88 and 30/70 for SCP-2% and SCP-5%, respectively. All experimental groups were isonitrogenous (45% crude protein) and isolipidic (18% crude lipid), with a P content of 0.8% (Table 1). Additionally, the diets contained denatured egg albumin (Vicens i Batllori S.L., Girona, Spain) as the sole protein source. The basal diet contained negligible amounts of P (0.02%).

Before formulating the diets, each ingredient was individually weighed and analyzed in triplicate, then mixed. Subsequently, the diets were prepared using a cooking-extrusion process with a semi-industrial twin screw extruder (CLEXTRAL BC-45, St. Etienne, France) at the UPV facilities. The processing conditions were as follows: a pressure of 4–5 MPa, a temperature of 110 °C and a screw speed of 100 rpm. Finally, the composition of all experimental diets was analyzed, also in triplicate, and are reported in Table 1.

### 2.3. Digestibility Trial

To obtain the feces, a Latin square experimental design of 4 treatments × 4 tanks × 4 trials was followed (consisting of four trials or periods, and in each of them, four tanks were fed with one of the feeds, so that the fish in all the tanks were fed with the two experimental feeds) [25,26,27]. Each collection period or trial lasted 14 days, 7 days of adaptation to the new diet without feces collection and 7 days collecting feces. The feces collection was performed in a settling column (water flow rate: 8.5 L/min, average density: 12 g/L) supplied with filtered freshwater.

For each experimental group, this procedure was repeated four times (n = 4). To reduce the tank effect, the tank location was changed in each repetition. Every day, fish were fed by hand until apparent satiation. The feeding rate was once per day at 10.00 a.m. from Monday to Saturday, with a starvation day on Sunday. Pellets were distributed slowly, allowing all fish to eat. Before feeding, the remaining uneaten feed was collated and feces were collected from trays, pooled by tank and frozen at −20 °C. At the end of the experimental period, feces collected from each tank were freeze-dried, prior to analysis. Before changing diets, the fish were starved for 24 h to help the ejection of the previous diet.

### 2.4. N and P Excretion Estimation

The excretion of ammonia and P was estimated as a function of the difference between the concentration of ammonia/P dissolved in a given time. The tanks were isolated for 20 min every two hours, closing the water and aeration of the tanks, where ammonia/P could only be accumulated by fish excretion.

Feeding was carried out at a restricted rate, according to the average weight of the fish and at water temperature, and the ration per tank was calculated according to the biomass of each fish. The same experimental design was followed as that described in the digestibility test.

Total ammonia N (N-TAN) and P excretions were calculated at each given time using the following equation:

Excretion (mg nutrienth)=((Final nutrient (mg)t0−Initial nutrient (mg)t1)time (h)t1)

The results of ammonia and P excretions were used to calculate the average hourly and/or daily excretion of ammonia/P and kg of fish. The initial and final amount of nutrient (N-TAN or P) was calculated by multiplying the volume of water in the tank by the concentration of N-TAN or P measured in mg/L.

### 2.5. Chemical Analyses and Calculations

Fish diets, feed ingredients and feces were analyzed based on the Association of Official Analytical Chemists (AOAC) [28] procedures: dry matter, official method 934.01 (105 °C to constant weight); crude protein, official method 990.03 (analyzed by the direct combustion method DUMAS using LECO CN628, Geleen, the Netherlands); crude lipid, official method 920.39 (extracted with methyl-ether using ANKOM^XT10^ Extractor, Macedon, NY, USA); and ash, official method 942.05 (incinerated at 550 °C for 5 h). All analyses were performed in triplicate.

An atomic absorption spectrophotometer (Perkin Elmer 3300, Perkin Elmer, Boston, MA, USA) was used for the analysis of Cr in feces and feed after acid digestion of the sample with HNO_3_ 1.5 N + KCl 0.38% [29].

The concentration of N-TAN in water was measured by the indophenol method after the addition of phenol, nitroprusside, sodium citrate and alkaline sodium dichloroisocyanurate (NaDTT). After 6 h, the absorbance of the compound was measured at a wavelength of 640 nm using a T60V UV-VIS spectrophotometer (PG Instruments, Leicester, UK).

Apparent digestibility coefficients (ADC_diet_, %) of N and P (%) were calculated using the following formulae described previously by [30]:

ADCdiet(%)=100−(Markerdiet×NutrientfaecesMarkerfaeces×Nutrientdiet)

In this equation, the terms Marker_diet_ (%) and Marker_feces_ (%) represent the marker content (Cr) of the diet and the feces, respectively, and Nutrient_diet_ (%) and Nutrient_feces_ (%) are the nutritional parameters of concern (e.g., N or P) in the diet and the faces, respectively.

### 2.6. Predicted Digestibility

Due to the critical importance of being able to distinguish between the different phosphates with higher bioavailability in each species, a predictive program has been developed considering the different chemical species that compound each phosphate, by means of different chemical parameters. Each of these chemical species has been assigned a digestibility weighting value based on previous in vivo studies [31,32,33,34,35]. The combination of the chemical balance with the weightings leads to Predictive Equations for Digestibility Comparison (EPCD) of commercial phosphates. In vivo digestibility estimation requires specialized settings, expensive operating costs and a high number of fish, and it is difficult to perform and achieve the desired experimental working conditions [36]. In this regard, EPCD can help to successfully evaluate the effect of diets on nutrient digestibility providing very reliable information.

From a chemical point of view, the EPCD predictive digestibility technology developed by Global Feed, S.L.U. (Tervalis group, Huelva, Spain) is based on the analysis of a multitude of chemical parameters, which are directly correlated with the presence of different chemical species that make up the phosphates on the market. These analyses are:

▪ Percentage of P, Ca and Na: determination of the composition of the product.
▪ Solubility in water: above 80% correlates with MCP, while solubility around 50% is due to the presence of MDCP.
▪ Solubility in alkaline ammonium citrate: almost completely dissolves DCP but not TCP, which remains insoluble.
▪ Solubility in 2% citric acid: difference in TCP (> 95%).
▪ Solubility in neutral ammonium citrate: good correlation with bioavailability.
▪ Percentage of CaCO_3_: presence of impurities and type of phosphate.
▪ Humidity: allows for quantification of free water.
▪ Loss 200–250 °C: estimate of hydration water.
▪ Ca/P ratio: estimation of the presence of different phosphates, as well as their composition.

Collecting the information described in previous studies [12,13,20,37,38,39,40,41,42], the parameters of the predictive equation have been modified and it has been estimated that, in the current test carried out on rainbow trout, the expected values will be in the proportion shown in Table 2. The predictive digestibility has been calculated by establishing the different chemical species in each inorganic phosphate and assigning a digestibility value based on bibliographic data such as those referred to above.

### 2.7. Statistical Analyses

Prior to analysis, the normal distribution was checked through a Kolmogorov–Smirnov test, while the homogeneity of variances using a Levene test. N-TAN and P values were measured as dissolved N concentration (mg N-TAN or P/L) and were transformed into mass units (mg N-TAN or P) to calculate ammonia excretion. The data on ammonia excretion are presented as produced ammonia N (mg N-TAN or P) divided by fish biomass (kg) and time (h). Two series of data are presented according to the approach of the trials. Firstly, the different values of N-TAN and P excretion that occur according to the time of day are graphed, where the daily variation between treatments can be observed. Secondly, all measured N-TAN and P consumption and daily excretion values were combined for statistical analysis.

Univariate ANOVAs were performed to determine the significant differences between treatments, using a Newman–Keuls test for comparison between means for a confidence interval greater than 95%. All statistical analyses were performed using Statgraphics^®^ Centurion v.XVII.II for Windows^®^.

## 3. Results

### 3.1. Effect of P Inorganic Sources on Digestibility

The digestibility data from diets and feces are shown in Table 3. ADC values of P were markedly influenced by inorganic P sources, with MAP and SCP-2% registering higher digestibility (92.26 and 90.08%, respectively) than SCP-5% and MCP sources (75.21 and 71.11%, respectively).

Fish fed an SCP-2% diet presented the highest values (92.80% and 93.07%) of protein and energy digestibility, while fish fed the SCP-5% and MCP diets registered statistically lower levels.

**Figure 2:**
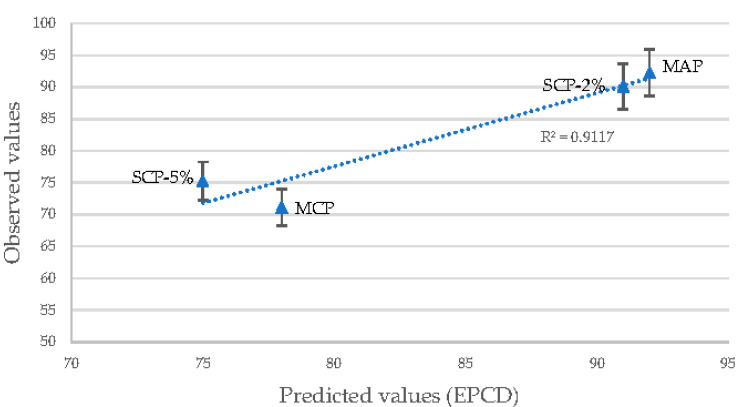
Relationship between the predicted and observed P digestibility values.

In Figure 2, the relationship between the predicted and observed values is shown. Values predicted for MAP and SCP-2% are very similar to those obtained in vivo, although in the case of inorganic phosphates which obtained lower P digestibility, the predicted values differ slightly.

### 3.2. Effect of Inorganic Phosphates on N and P Excretion

**Figure 3:**
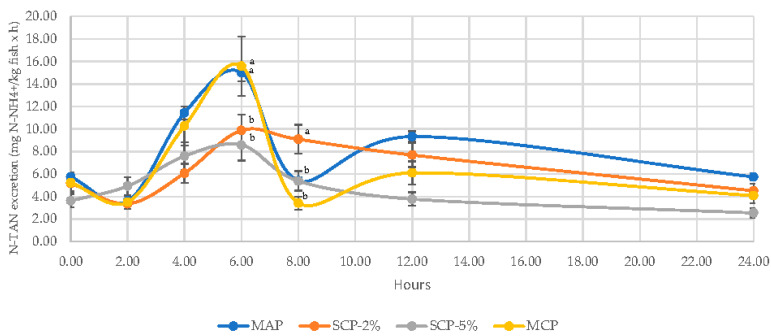
Daily pattern of soluble total ammoniacal N excretion rate (expressed in mg of N-NH_4_^+^ per kg of fish biomass and hour) of rainbow trout fed four inorganic P sources. Each point represents the average (±SE) of four tanks. Different superscripts indicate statistically significant differences with *p* < 0.05. A Newman–Keuls test was used to compare the means. The amount of N excretion was determined based on N-TAN concentration in water every two hours after feeding over a 24-h period.

The N excretion patterns are shown in Figure 3. In spite of dietary treatment, the peak N excretion occurred at 6 h post meal ingestion, decreasing afterwards until basal levels at 18 h after feeding. If the dietary treatment is considered, at 6 h, N excretion was lower in the SCP-5% and SCP-2% diets (8.57 and 9.87 mg N-NH_4_+/kg fish × h, respectively); however, at 8 h after feeding, SCP-2% maintained higher levels than the rest (9.09 mg N-NH_4_+/kg fish and h) (Figure 3).

**Figure 4:**
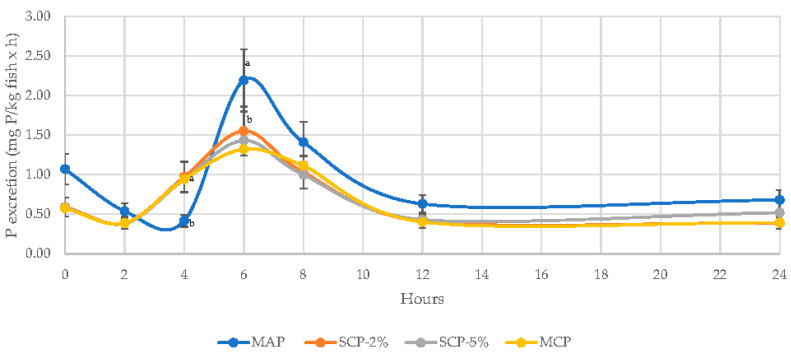
Daily pattern of soluble total P excretion rate (expressed in mg of P per kg of fish biomass and hour) of rainbow trout fed four inorganic P sources. Each point represents the average (±SE) of four tanks. Different superscripts indicate statistically significant differences with *p* < 0.05. A Newman–Keuls test was used to compare the means. The amount of P excretion was determined based on the P concentration in water and the water flow every hour after feeding over a 24-h period.

As N excretion, regardless of inorganic P sources, the peak P excretion occurred at 6 h post meal ingestion, followed by a decrease down to basal levels at 12 h after feeding (Figure 4). Fish fed MAP as P inorganic sources significantly presented the highest P excretion at 6 h post feeding (2.19 mg P/kg fish and h).

### 3.3. Total N and P Losses

**Figure 5:**
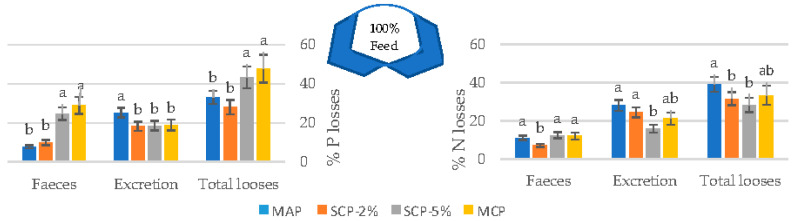
Nutrient mass budget for N and P expressed as a percentage of the amount of dietary nutrient provided to fish. Each point represents the average (±SE) of four tanks. Different superscripts indicate statistically significant differences with *p* < 0.05. A Newman–Keuls test was used to compare the means.

Considering both soluble and solid N and P discharged fractions, N and P wastes for each inorganic P source expressed per percentage of feed intake have been evaluated as shown in Figure 5.

Fish fed a diet supplemented with MAP released a higher amount of P in its dissolved form to the water in comparison with the rest of inorganic P sources (Figure 5), although the total losses were lower in MAP and MCP (33.02 and 28.13%, respectively) than in SCP-5% and MCP sources (43.35 and 47.83%, respectively).

In the case of N, SCP-2% showed the lowest N discharge by fecal losses (7.2%), but in the case of N excretion, SCP-5% provided lower values (15.8%) than MAP (28.0%). Therefore, SCP-2% and SCP-5% showed the lowest total waste of N (31.54 and 28.25%, respectively) and MAP showed the highest values (39.01%) of total N intake.

## 4. Discussion

The N and P digestibility as well as the excretion of commercial inorganic phosphates has been evaluated assessing its nutritional and also its environmental value for fish diets. Both treatments, MAP and SCP-2%, showed higher values than SCP-5% and MCP in terms of P digestibility (92 and 90%, respectively). If it is considered that the only P source comes from diet, we can state that the results obtained are also very similar to those shown in the study carried out by Morales with rainbow trout [20], who also reported P digestibility coefficients above 90%. In Japanese amberjack (*Seriola quinqueradiata*), with a similar feed, formulation greater than 90% of P digestibility was obtained [37].

Compared with the digestibility results in other species, the values of the digestibility of different inorganic phosphates were similar to those obtained in the present experiments. The MSP and DCP values were in concordance with those obtained for shrimp [43] but considerably lower than the values of 90–98% and 46–71%, respectively, reported for fish [40]. The 73% value for the MCP is higher than the 49% reported for shrimp [43]. These differences in digestibility are mainly due to the different degrees of solubility between phosphates, where, for example, MCP has a much higher solubility to water (>85%) than DCP (<25%). This difference in solubility is very relevant, since it reflects the ability of P to solubilize at neutral pH, simulating the intestine (absorption of P). This fact is even more important in fish, since they lack a true stomach in which a first stage of solubilization begins prior to digestion.

The needs of P in the diet depend on several factors [39], including the bioavailability of the element, food intake, the requirement for new tissue synthesis and the amount of endogenous loss, among others; these factors being dependent on the life cycle and size, and even environmental factors such as salinity and temperature. Commercial feed usually contains from 10 to 15 g P/kg with varying P digestibility content, which depends on ingredient quality and P sources used in aquafeeds. If both the level of dietary P and P ADC are taken into account, the levels of digestible P would be 0.923 and 0.90%, respectively, for MAP and SCP-2% treatments, which would cover the needs of digestible P since around 0.8% is recommended in salmonids [5,44]. Nevertheless, it is remarkable that the SCP-2% diet registered higher ADCs in terms of N and energy. The improved digestibility of SCP-2% requires further investigation, since the sources of protein and lipids have been at the same proportions in both treatments. It is difficult to explain the differences observed in the present experiment regarding N digestibility, because the protein source was the same in all treatments and only the inorganic phosphate source differed between diets. One possible explanation could be the differential buffering capacity (BC) of inorganic phosphates, which could affect the pH, and consequently, modulate digestive enzyme activities, and therefore, raising the protein and mineral content [45]. In previous studies, BC was tested in ingredients used for animal and fish diets. For example, cereals reported lower buffering capacity than plant protein meals [46]. On the other hand, plant protein registered lower capacity than fish meal and gluten [46]. Thus, the ingredients used in diets can modify feed BC, as has been reported in sea bass (*Dicentrarchus labrax*) [47], Atlantic salmon (*Salmo salar*) [48] and sea bream (*Sparus aurata*) [49,50]. In the current experiment, the buffering capacity of SCP-2% was significantly lower (866 meq HCl/kg) than the rest of the inorganic phosphates (1966 meq HCl/kg), which might imply an advantage of including this additive in fish diets, as has been reported [51].

A high correlation (>0.9) was obtained between the results of the in vivo P digestibility assay and the estimated results using the EPCD index. This index could become a useful tool to formulate practical diets. Nevertheless, it is noteworthy that the only factor not taken into account in the EPCD index is the variability stemming from the different particle sizes of phosphate; therefore, an inclusion of this factor might be needed for products with a different granulometry or the comparison only of products with similar textural properties.

The P excretion into the water system provided similar values independent of the diet, with a maximum at 6 h after feeding, similar to previous studies [16]. Additionally, the daytime pattern of soluble P is in accordance with other studies in trout fed semi-purified diets [52]. On the other hand, the MAP diet provided higher P excretion at almost all sampling points. Some researchers have applied the classical concept of balance study to determine the effect of diet on the fecal excretion of soluble P. Using this concept, the authors indicate that when fish consume excess available P, the excess P is mainly eliminated through the gills and kidneys as non-fecal soluble P [52,53,54]. Excretion of soluble non-fecal P also depends primarily on P sources, i.e., when fish ingest an excess of highly available inorganic P, excretion of soluble non-fecal P should increase compared to digestion from sources of low P availability [52]. Therefore, differences in P excretion observed in the present study might be due to the available P contained in the diets. Here, the excretion of soluble P per fish is generally independent of dietary P up to a certain level of dietary P, but then increases considerably above this level. This suggests that excretion of soluble P occurs when the available P intake is above levels sufficient for retention and/or when the mechanisms of intestinal absorption and renal reabsorption are saturated. In the rainbow trout intestine, there is a sodium-dependent inorganic P carrier that is closely regulated by dietary P [16]. Intestinal absorption rates of inorganic P decrease as dietary P levels increase, suggesting that the effectiveness of phosphate transport systems in retaining dietary P may decrease when dietary P levels are very high [55]. Vitamin D3, which improves intestinal absorption of inorganic P and renal reabsorption in birds and mammals, has no effect on the intestinal absorption of inorganic P in diets fed for trout containing sufficient amounts of P [56]. A collection of data from other studies confirms that the production of soluble P per kg of fish is a linear function of dietary P and presumably independent of P source [52]. Soluble P is the most flexible component of effluent P. There is no excretion of soluble P at low levels of dietary P, but soluble P becomes the main route of excretion as the available P concentration increases above hypothetical levels of P requirements for the species. In fact, in diets containing less than 0.22 ± 0.03% available P, there is no production of soluble P [52] and excretion increases at a rate of 6.6 ± 0.67 mg/day/kg for every 0.1% increase in available dietary P [54], which is roughly in line with the data obtained.

Analyzing the overall data on daily excretion rate, the results corroborate that fish fed MAP feed had significantly higher N excretion, probably due to the ammonium content of MAP, as has been reported in previous studies [20]. These excretion data have been calculated by way of the difference of N-TAN concentration in the water; thus, based on the results obtained, it can be stated that there is a higher concentration of N-TAN in the water system in the MAP experimental group. This excess excretion must be considered when sizing biofilters in fish recirculation systems, or in the case of open systems, since it will mean a greater release of ammoniacal N into the natural environment, which will lead to greater eutrophication of the water. In the case of soluble P, there were no significant differences in the MAP treatment with respect to SCP-2% and MCP.

Few studies have registered an increase of N residues from inorganic P dietary supplement containing indigestible N, such as MAP, compared to other phosphate sources. Morales [20] reported that higher discharge of ammonium ions directly through the feces, when MAP is used as a dietary supplement, could lead to an overload of undesirable reduced N fraction dissolved in aquaculture recirculation systems or other high intensity systems. Therefore, in addition to optimizing dietary P, the use of diets with an optimal level of digestible N will become another key to sustainable aquaculture.

Considering fecal and soluble P losses, even though data on P retention are not available, it was possible to carry out an approximation of P and N losses in each of the treatments. As can be seen, in general, fish fed with MAP show a higher percentage of N losses than those fed with SCP-2% and SCP-5%, under the experimental conditions tested. Sugiura et al. [6] found that 38% of the total P fed was not assimilable and the P excretion represented up to 20% of total P ingested by trout, reporting P losses [57] similar to those obtained only with MCP.

Considering N and P as the most relevant nutrients for inducing water eutrophication [57], it would be relevant to minimize these components into the column water. Currently, there are a wide range of commercial inorganic phosphate available; however, the most used in aquafeed are the monoammonium, monocalcium and monosodium phosphates due to their good availability in fish, although the new formulation (SCP-2%) can improve the wastes generated in the aquaculture production, which is crucial to improve the environment.

## 5. Conclusions

The SCP-2% source (AQphos+) presents a phosphorus digestibility comparable to MAP (without significant difference at a statistical level), but with lower P and N excretion, and thus, it is more environmentally friendly. Therefore, SCP-2% as a phosphorus source is more advantageous from a nutritional, environmental and industrial point of view (biofilters and recirculation systems in fish farms).

## Figures and Tables

**Table 1:** Formulation and proximate composition of the experimental diets.

Experimental Diets	MAP	SCP-2%	SCP-5%	MCP
Ingredients (g/kg)				
Albumin ^1^	450	450	450	450
Fish oil ^2^	180	180	180	180
Starch ^3^	200	200	200	200
Maltodextrin ^4^	3	108.2	107.9	107.1
Feeding stimulant ^5^	5	5	5	5
Cr_2_O_3_	5	5	5	5
Monoammonium phosphate ^6^	31.7			
Monosodium/monocalcium phosphate (2%) ^7^		31.8		
Monosodium/monocalcium phosphate (5%) ^8^			32.1	
Monocalcium phosphate ^9^				32.9
Multivitamin and minerals mix ^10^	20	20	20	20
Analyzed composition (% dry weight)				
Dry matter (% DM)	95.86	96.83	97.50	96.88
Crude Protein (% CP)	45.80	45.41	45.44	45.75
Crude Lipids (% CL)	17.83	18.01	18.10	17.95
Crude phosphorous (% P)	0.84	0.87	0.85	0.82
Ash (%)	4.96	5.98	5.75	5.12
Nitrogen (% N)	7.33	7.27	7.27	7.32
Carbon (% C)	42.9	43.1	43.2	42.7
Calculated values				
Gross Energy (kJ/g) ^11^	22.21	22.32	22.36	22.11

^1^ Albumin (93.4% DM; 97% CP). Vicens I Batllori S.L. (Girona, Spain). ^2^ Fish oil. INDUSTRIAS AFINES, S.L., (Pontevedra- Spain). ^3^ Starch. Distribucines exclusivas de pasteleria SA, (Valencia, Spain) ^4^ Maltodextrin; (96% carbohydrates). Productos Pilarica S.A. (Valencia, Spain). ^5^ Feeding stimulants: Glutamic acid, Proline, Methionine Arginine and Histidine (0.2, 0.2, 0.2, 0.3 and 0.1%, respectively); Guinama^®^. (Valencia, Spain). ^6^ NH_4_H_2_PO_4_, inorganic phosphate, Bolifor MAP, containing 25.13% of P in dry matter; Yara S.L. (Helsingborg, Finland). ^7^ NaH_2_PO_4_/Ca(H_2_PO_4_)_2_·H_2_O (2%), inorganic phosphate, AQphos+, containing 24.95% of P in dry matter; Global Feed, S.L.U. (Huelva, Spain). ^8^ NaH_2_PO_4_/Ca(H_2_PO_4_)_2_·H_2_O (5%), inorganic phosphate, prototype product, containing 25.22% of P in dry matter; Global Feed, S.L.U. (Huelva, Spain). ^9^ Ca(H_2_PO_4_)_2_·H_2_O, inorganic phosphate, prototype product, containing 24.31% of P in dry matter; Global Feed, S.L.U. (Huelva, Spain). ^10^ Multivitamin and minerals mix (values are g/kg except those in parenthesis): Premix: 25; Choline, 10; DL-α-tocopherol, 5; ascorbic acid, 5; (PO_4_)_2_Ca_3_, 5. Premix composition: retinol acetate, 1,000,000 IU/kg; calciferol, 500 I/kg; DL-α-tocopherol, 10; menadione sodium bisulfite, 0.8; thiamine hydrochloride, 2.3; riboflavin, 2.3; pyridoxine hydrochloride, 15; cyanocobalamin, 25; nicotinamide, 15; pantothenic acid, 6; folic acid, 0.65; biotin, 0.07; ascorbic acid, 75; inositol, 15; betaine, 100; polypeptides, 12. ^11^ Gross energy (kJ/g) = [51.8 × (%C/100)) − (19.4 × (%N/100)]. Calculated according to Brouwer [24].

**Table 2:** Predictive values of P digestibility (%) obtained by Predictive Equations for Digestibility Comparison (EPCD) of inorganic phosphates adapted for fish.

Inorganic Source	EPCD Value (%)
MAP	92
SCP-2%(AQphos+)	91
SCP-5%	75
MCP	78

**Table 3:** Results of apparent digestibility coefficients (ADC, %) by treatment *.

	MAP	SCP-2%	SCP-5%	MCP
ADC P	92.26 ^a^ ± 2.19	90.08 ^a^ ± 1.26	75.21 ^b^ ± 2.51	71.11 ^b^ ± 1.89
ADC protein	88.98 ^b^ ± 2.81	92.80 ^a^ ± 1.56	87.58 ^b^ ± 5.01	87.91 ^b^ ± 2.81
ADC energy	84.68 ^b^ ± 6.86	93.07 ^a^ ± 1.45	78.51 ^c^ ± 5.02	75.99 ^c^ ± 2.93

* ADC: Apparent digestibility coefficient for each diet (%). The data show the mean of the four tanks following a Latin square experimental design (4 × 4) ± standard deviation of the mean (±SE). Different superscripts indicate statistically significant differences with *p* < 0.05. A Newman–Keuls test was used to compare the means.
